# Novel Aspects of Renal Magnesium Homeostasis

**DOI:** 10.3389/fped.2018.00077

**Published:** 2018-04-09

**Authors:** Paula Giménez-Mascarell, Carlotta Else Schirrmacher, Luis Alfonso Martínez-Cruz, Dominik Müller

**Affiliations:** ^1^CIC bioGUNE, Bizkaia Science and Technology Park, Derio, Spain; ^2^Department of Pediatric Gastroenterology, Nephrology and Metabolism, Charité – Universitätsmedizin Berlin, Berlin, Germany

**Keywords:** magnesium, crystallography, CNNM2, kidney, genetics

## Abstract

Magnesium (Mg^2+^) is indispensable for several vital functions, such as neurotransmission, cardiac conductance, blood glucose, blood pressure regulation, and proper function of more than 300 enzymes. Thus, Mg^2+^ homeostasis is subject to tight regulation. Besides the fast and immediate regulation of plasma Mg^2+^, a major part of Mg^2+^ homeostasis is realized by a concerted action of epithelial molecular structures that tightly control intestinal uptake and renal absorption. This mechanism is provided by a combination of para- and transcellular pathways. Whereas the first pathway provides the organism with a maximal amount of vital substances by a minimal energy expenditure, the latter enables controlling and fine-tuning by means of local and regional regulatory systems and also, hormonal control. The paracellular pathway is driven by an electrochemical gradient and realized in principal by the tight junction (TJ), a supramolecular organization of membrane-bound proteins and their adaptor and scaffolding proteins. TJ determinants are claudins (CLDN), a family of membrane spanning proteins that generate a barrier or a pore between two adjacent epithelial cells. Many insights into molecular mechanisms of Mg^2+^ handling have been achieved by the identification of alterations and mutations in human genes which cause disorders of paracellular Mg^2+^ pathways (CLDN10, CLDN14, CLDN16, CLDN19). Also, in the distal convoluted tubule, a basolateral protein, CNNM2, causes if mutated, familial dominant and also recessive renal Mg^2+^ wasting, albeit its true function has not been clarified yet, but is assumed to play a key role in the transcellular pathway. Moreover, mutations in human genes that are involved in regulating these proteins directly or indirectly cause, if mutated human diseases, mostly in combination with comorbidities as diabetes, cystic renal disease, or metabolic abnormalities. Generation and characterization of animal models harboring the corresponding mutations have further contributed to the elucidation of physiology and pathophysiology of Mg^2+^ disorders. Finally, high-end crystallization techniques allow understanding of Mg^2+^ handling in more detail. As this field is rapidly growing, we describe here the principles of physiology and pathophysiology of epithelial transport of renal Mg^2+^ homeostasis with emphasis on recently identified mechanisms involved.

## Introduction

Magnesium (in its ionized and biologically active form: Mg^2+^) belongs to the group of alkaline earth metals and is the second most abundant intracellular divalent cation. It is the eleventh most abundant element by mass in the human body. Mg^2+^ is indispensable for several vital functions, such as neurotransmission, cardiac conductance, blood glucose control, and blood pressure regulation. In muscle, Mg^2+^ regulates contraction by antagonizing calcium (1–4). Mg^2+^ has also been identified as a second messenger, e.g., in T-Cells, where mutations in the gene MAGT1 have been linked to human immunodeficiency. There, mutations disable transient Mg^2+^ influx induced by the activation of the T-cell receptor (5).

Adenosine triphosphosphate (ATP) is the major source of cell energy, and must bind Mg^2+^ in order to be biologically active. The resulting complex, Mg^2+^-ATP is vital for the stability of all polyphosphate compounds in cells, including those associated with synthesis of DNA and RNA. More than 300 enzymes are dependent on Mg^2+^ for their biocatalytic function, including those that utilize or synthesize ATP, or those that use other nucleotides to synthesize DNA and RNA (6). In plants, Mg^2+^ is the central ion of chlorophyll and, therefore, vital for photosynthesis. In higher organisms, hemoglobin, the essential O_2_ carrier, has high structural similarities with chlorophyll but here, Fe^2+^ replaced Mg^2+^ as the central ion. Magnesium is an essential mineral nutrient (i.e., element) and is present in every cell type and in every organism. In the blood and serum, Mg^2+^ is mostly bound to serum albumin (like the most abundant divalent cation, Ca^2+^) and stored in muscle fibers and in bone. The biologically active form is the ionized form and dietary sources rich of magnesium are plants [Almonds, Cashews, Cocoa, Pumpkin Seeds, Spinach, and Fish (Halibut, Mackeral)]. Clinically, deficiency of Mg^2+^ causes nausea, appetite loss, fatigue, and general weakness. At a later stage, patients present with numbness, tingling, muscle cramps, cerebral seizures, and cardiac arrhythmias. Moreover, Mg^2+^ deficiency can lead to hypocalcemia and hypokalemia (7). On the other hand, little is known about the consequences of hypermagnesemia as can be encountered, e.g., in patients with end stage renal disease (8). However, Mg^2+^ complexes are involved in the development of vascular calcifications, a major cause of morbidity and mortality of patients with chronic kidney disease (9).

Compared to Ca^2+^ storage (around 1000 g in adults), the whole body content of Mg^2+^ only sums up to 20 g. In addition, Mg^2+^ stores are not as readily accessible as Ca^2+^ stores by, e.g., PTH action. Therefore, the organisms’ intra- and extracellular Mg^2+^ levels are kept tightly within narrow limits. Likewise, the available, ionized, biologically active and relevant form (Mg^2+^) is dependent on a more or less a continuous nutritional supply. Mg^2+^ serum concentrations in adults range from 0.7 to 1.1 mmol/l, while newborns and toddlers can have lower limit serum concentrations (e.g., from 0.45 mmol/l on). The daily need of Mg^2+^ in adults is around 300–350 mg/day and fractional intestinal uptake varies depending on the amount of intake. Fine et al. found that intestinal Mg^2+^ absorption increases with intake but also that fractional Mg^2+^ absorption fells progressively (from 65% at low to 11% at high intake) (10). The authors described the intestinal absorption by an equation containing a hyperbolic function plus a linear function. They concluded that Mg^2+^ absorption must, therefore, be realized by a twin-mechanism that simultaneously reaches an absorptive maximum, and in addition a mechanism that endlessly absorbs a defined fraction (7%, as the authors concluded) (10). Although the molecular mechanisms have not been identified at that time, their and the findings of other groups later, predicted the coexistence of two distinct mechanisms of Mg^2+^ absorption, the trans- and paracellular transport in the intestine. Moreover, as we know nowadays, the same holds true for the kidney.

**Figure 1:**
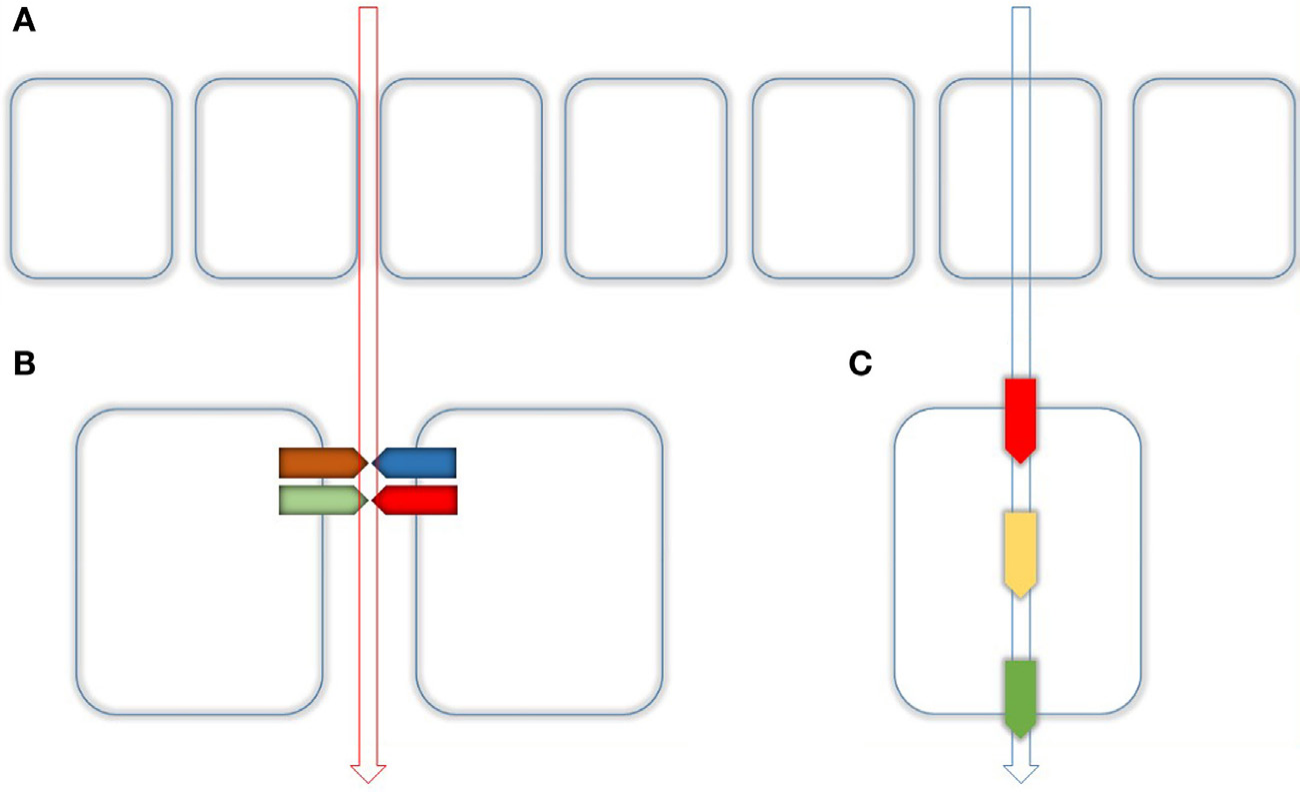
Routes of epithelial transport. **(A)** Epithelial transport is performed by two different routes. Paracellular (left) and transcellular (right) as indicated by the vertical arrows. **(B)** The paracellular transport is realized through the tight Junction (TJ) which is composed of several Proteins mainly by Claudins. Together with their adaptor and scaffolding proteins, they make up as hetero- or homodimers dimers the main part of the TJ. **(C)** Paracellular transport is realized by apical uptake (red), intracellular buffering and transport (yellow), and basolateral extrusion (green).

In both organs, the intestine and the kidney, Mg^2+^ is absorbed *via* paracellular as well as by transcellular routes. Whereas the first pathway is in principal driven by an existing electrochemical gradient, the latter requires energy-consuming mechanisms in order to conduct apical uptake, buffering, transport, and basolateral extrusion (Figure 1). The paracellular route guarantees the organism to retrieve a considerable amount of vital substances with a minimal energetic expenditure. On the other side, the energy-consuming transcellular route provides a fine-tuning at the several steps of transport and is, therefore, subject to precise local, regional, or global regulation. This can be realized by hormones such as 1,25(OH)D_2_ or parathyroid hormone according to the basic but also the actual needs of the organism (e.g., growth).

In the kidney, the driving force of transepithelial Mg^2+^ absorption is provided by a continuously maintained electrochemical gradient between the apical (tubular fluid) and the basolateral (blood) compartment. This process is realized by a battery of transcellular (and apical to basolateral) and paracellular transport mechanisms. As a principle, paracellular transport mainly takes place where the transepithelial concentration gradient is high, i.e., the proximal jejunum in the gut and in the proximal tubule (PT) as well the thick ascending loop of Henle in the kidney. By contrast, transcellular transport takes place in the late ileum, colon, and the distal part of the renal tubule (11, 12).

## Magnesium Handling along the Nephron

After glomerular filtration, the non-protein-bound fraction, Mg^2+^ is absorbed along the nephron before it is finally lost irretrievable for the organism within the urine. Under normal conditions (Glomerular filtration rate >90 ml/min/1.73 m^2^), more than 95% of filtered Mg^2+^ in the pro-urine is reabsorbed along the tubular system by several coordinated transport processes (13). Besides their anatomical localization, regulatory mechanisms of renal Mg^2+^ homeostasis can also be classified by their involvement in the hierarchy of transport. Therefore, we classify here the mechanisms of Mg^2+^ transport and homeostasis according to their involvement in the network of Mg^2+^ handling (Table 1). The first category comprises proteins or elements that transport Mg^2+^ by itself, e.g., a direct Mg^2+^ transport mechanism. The second category includes proteins and mechanisms that regulate elements of category 1. The third category comprises members that influence Mg^2+^ handling in a more remote way, e.g., by regulating ions, other than Mg^2+^ but thereby influence Mg^2+^ handling. To the latter category belong also proteins that influence Mg^2+^ homeostasis, e.g., by causing polyuria and thereby a “wash out” of Mg^2+^ by reducing the time of contact of absorbing mechanisms with the corresponding substance (as can be seen by the treatment with furosemide). With this newly proposed classification, we aim to focus on the current knowledge on Mg^2+^ handling toward a more interactive model, the magnesiome, and the Mg^2+^ interactome.

**Table 1:** Protein classification based on the role played by each molecule in renal magnesium handling.

Category	Role in Mg^2**+**^ handling	Proteins
1	Direct transport of Mg^2+^	Claudin-16^a^; Claudin-19^a^; TRPM6^b^; CNNM2^c^^,^^b^; SLC41A3^c^^,^^b^
2	Regulation of a direct transporter/channel of Mg^2+^	Claudin-14^a^; EGF^b^; CNNM2^c^^,^^b^
3	Contribution to Mg^2+^ homeostasis by indirect means	NKCC2^a^, Claudin-10^a^; ROMK^a^^,^^b^; PCBD^a^^,^^b^, HNF1B^a^^,^^b^, Barttin^a^^,^^b^; Na^+^-K^+^-ATPase^a^^,^^b^/FXYD2a,b^a^^,^^b^; CaSR^a^^,^^b^; ClC-Kb^a^^,^^b^; Kir4.1^b^; Kv1.1^b^; NCC^b^

*^a^Proteins expressed in the TAL*.

*^b^Proteins expressed in the DCT*.

*^c^Proteins which role is under debate*.

**Figure 2:**
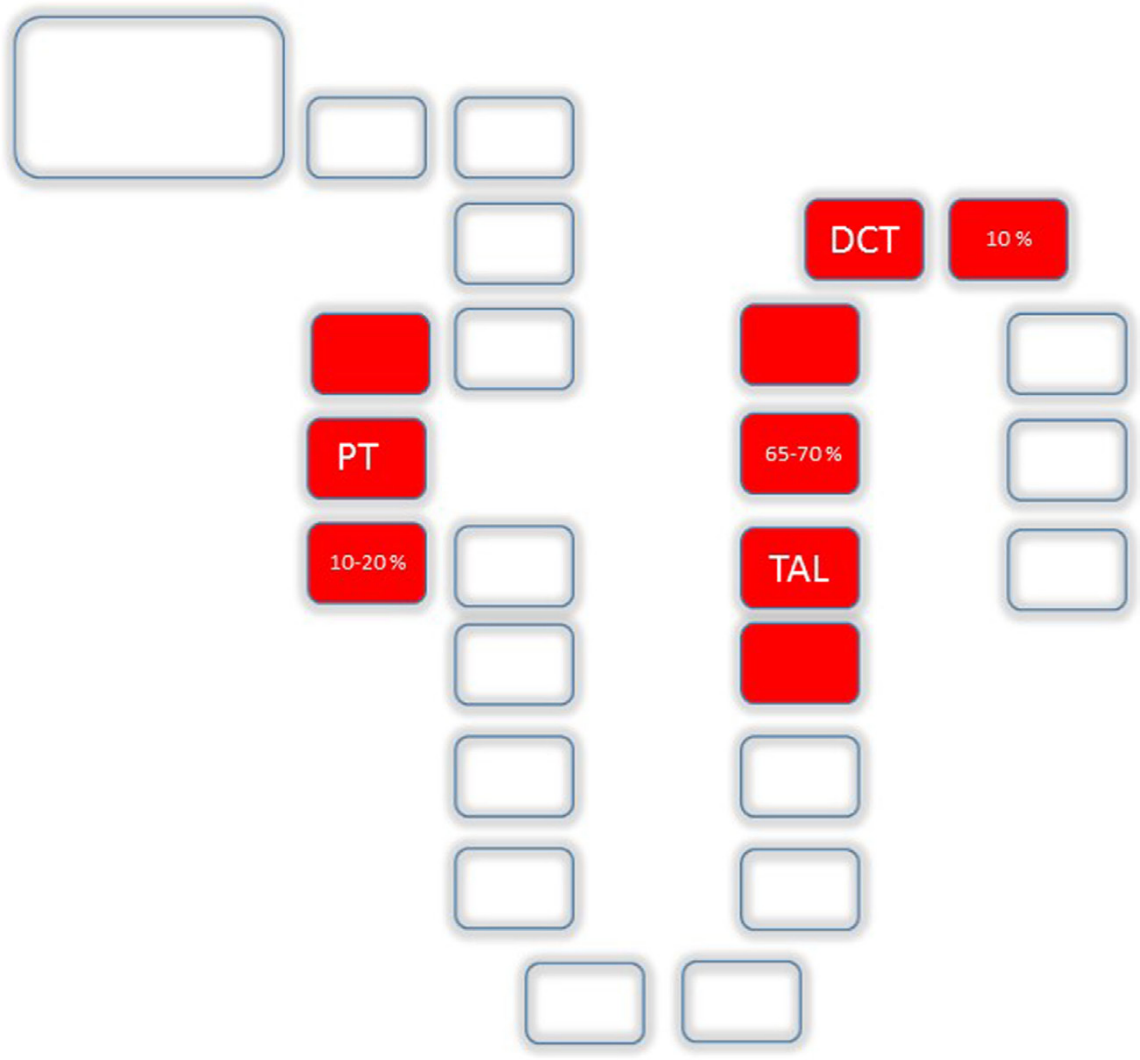
Mg^2+^ absorption along the nephron: Uptake along the nephron is realized by the proximal tubule (PT) (10–20%), the TAL (65–70%) and the distal convoluted tubule (DCT, 10%).

Anatomically, the major sites of renal Mg^2+^ reabsorption are, besides the PT (10–20%), the thick ascending loop of Henle (TAL, 65–70%) and the distal convoluted tubule (DCT, 10%) (Figure 2). Beyond the DCT, no significant mechanisms of Mg^2+^ absorption have been described so far (11, 12).

### PT and Thick Ascending Part of Henle’s Loop

**Figure 3:**
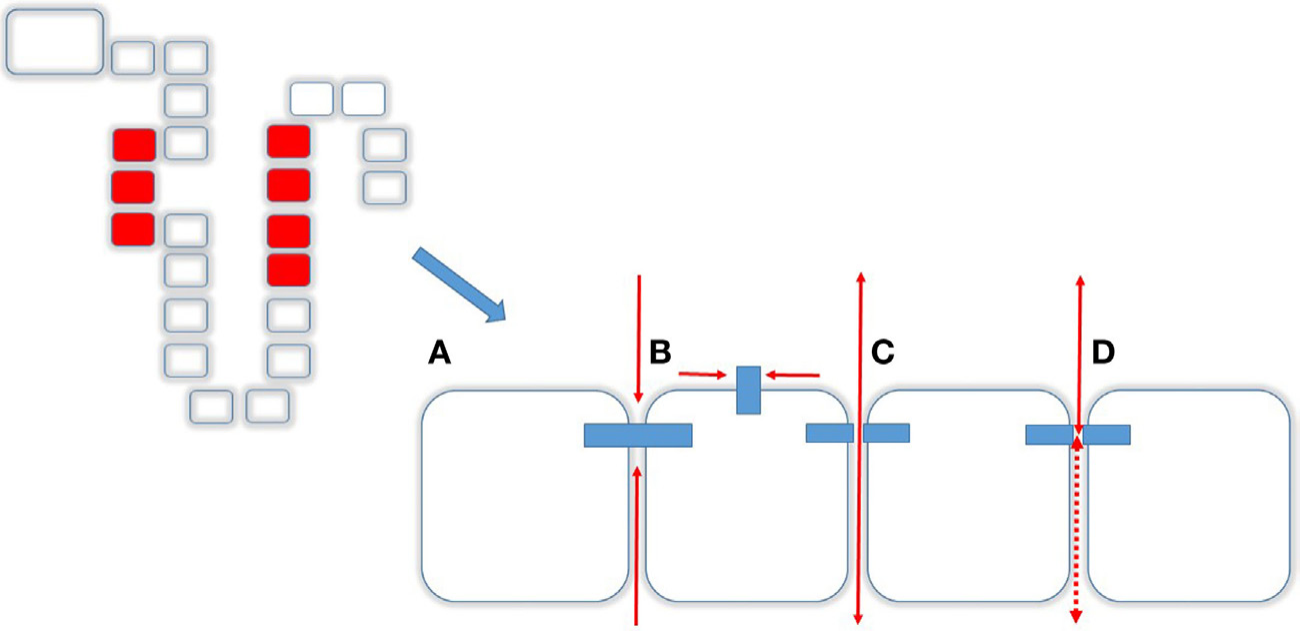
Transcellular transport. Tight junctions (TJs) and Caudins are responsible for different functions **(A)** Barrier, i.e., no transit in any direction, **(B)** Fence, enabling a cell apical-to-basolateral orientation, **(C)** Channel function, allowing molecules to pass from apical to basolateral and *vice versa*, and **(D)** depending on the composition of the TJ.

**Figure 4:**
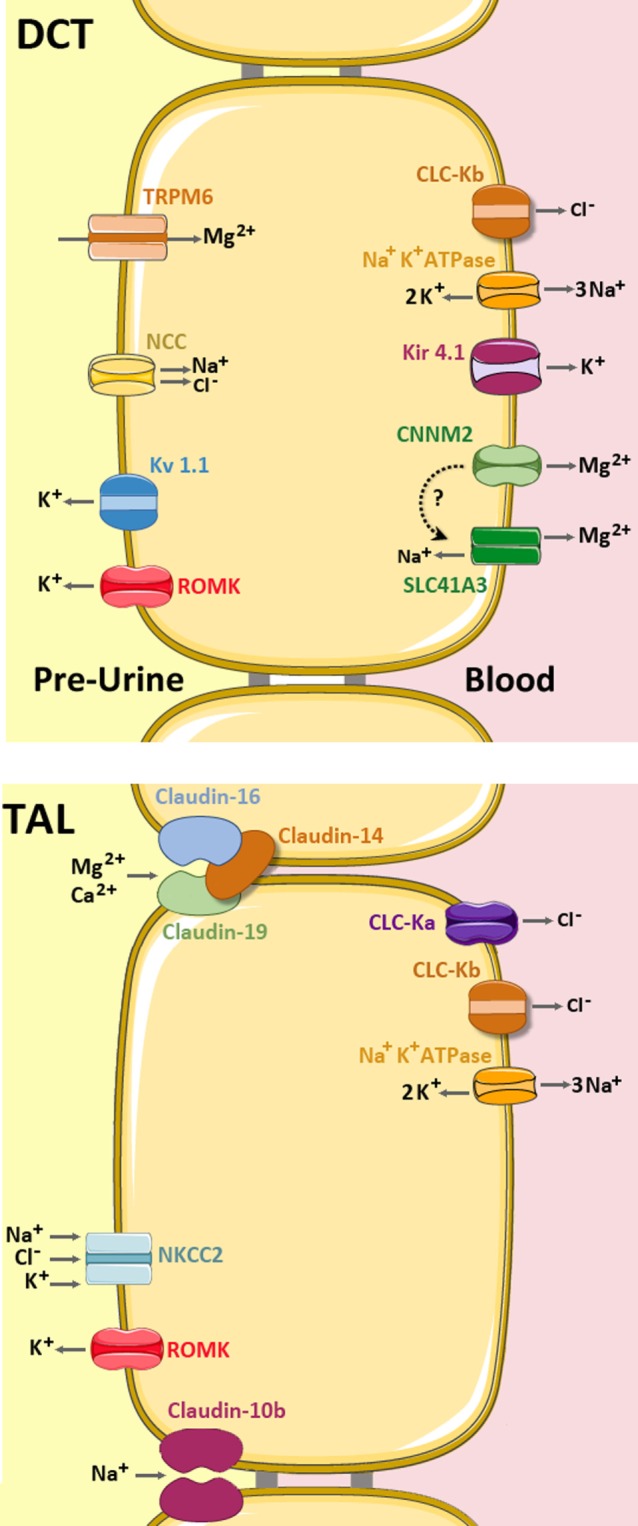
Mg^2+^ handling network along the TAL and distal convoluted tubule (DCT). In the TAL (bottom), Mg^2+^ and Ca^2+^ are absorbed *via* the permeable channel formed by Claudin-16 and Claudin-19, which is inhibited by Claudin-14 (light brown). Claudin-10b is a cation-selective channel with high preference for Na^+^. The apical force needed for Mg^2+^ reabsorption is created by the cooperative activities of the basolateral Na^+^ K^+^ ATPase pump, the Chloride channels ClC-Ka and ClC-Kb, and the apical Na^+^ K^+^ 2Cl^−^ cotransporter (NKCC2) and the K^+^ extruder, ROMK. (up) In the DCT, Mg^2+^ is mainly absorbed by transcellular routes. The Mg^2+^ ions enter into the cell through the apical TRPM6 channel and are then extruded by either CNNM2 and/or SLC41A3 (still under debate). The apical force needed for Mg^2+^ transport is created by the cooperative action of the basolateral Na^+^ K^+^ ATPase, the Cl^-^ channel ClC-Kb, the K^+^ transporter Kir 4.1 and the apical Na^+^ Cl^−^ cotransporter (NCC) and the K^+^ extruders, ROMK and Kv 1.1 (5, 27).

Mg^2+^ absorption in the PT and TAL occurs mainly *via* paracellular route and in the DCT the transcellular route (13). The paracellular route is mainly determined by the Tight Junction (TJ), a supramolecular organization of membrane-bound proteins and their intracellular adaptor- and scaffolding proteins. The major proteins of the TJ comprise Claudins (from lat. *claudere*: to seal), a protein family consisting of at least 24 members in Eukaryotes enabling the TJ to function as either (a) barrier, (b) fence, or (c) channel (Figure 3). The *Barrier* function of the TJ enables the organism to increase transepithelial resistance (TER; Ω × cm^2^) where needed. As such, the PT has a low resistance (6–10 Ω × cm^2^) whereas downstream, toward TAL (11–34 Ω × cm^2^) and the Collecting Duct (60 Ω × cm^2^; MDCK cells) TER is constantly increasing (14, 15). The bladder, in order to fulfill its function of a tight reservoir, has the highest epithelial resistance [>300 kΩ × cm^2^ (16); Figure 3A]. The *Fence* function of the TJ is a key element that contributes to the apical-to-basolateral orientation of epithelial cells. In order to guarantee a coordinated, regulated transcellular transport, membrane-bound proteins must be oriented stable at either apical or the basolateral side. This principal requirement of all epithelial tissues is mainly realized by the TJ (17). Likewise, the loss of the apical-to-basolateral orientation, i.e., the loss of polarity and even cell-to-cell contact is an early event in tumor development. Several claudins have been shown to be potential markers of gastrointestinal tumors and their progression (18). Moreover, other Claudins (e.g., Claudin-3) have been demonstrated to be involved, in men and animals in tumor invasiveness and in autoimmune disorders (18, 19). Also, several Claudins are vital for teeth development in mice and humans (20). A great step toward the determination of the role of Claudins has been achieved by the resolution of the crystal structure of the Claudin protein Claudin-15 (21, 22) (Figure 3B). Although their contribution to human disease has been demonstrated, proteins that directly interfere with Claudins have only been preliminarily characterized (23, 24). The *Channel* function of the TJ is crucial for ions (like Mg^2+^ and Ca^2+^) and H_2_0 absorption and is dependent on an existing electro-chemical gradient (17). The *Channel* function is realized by a surplus of one charge on one side of the epithelial layer than on the other side (e.g., apical vs. basolateral or vice versa) or on the surplus of a molecule or substance over the epithelial layer. Although the TJ does not possess pumps or antiporters, transport can nevertheless be selective by the variation and regulation of TJ composition and protein expression (25). Thus, the expression of different Claudins along epithelial tissues and their regulation and expression can determine Mg^2+^ spatial and temporal reabsorption (26) (Figure 4).

Although our knowledge on Mg^2+^ handling in the more distal nephron has increased significantly, little is known about Mg^2+^ transport in the PT, where it is believed to be transported by the paracellular way. There, terminus “bulk reabsorption” is used widely although this phenomenon has not been elucidated in detailed molecular or physiological context.

Bartter’s Syndrome, first described by the endocrinologist Frederick Bartter, is characterized by renal wasting of Na^+^, K^+^, and polyuria. Clinically, hyperaldosteronism in preterm borns, polyhydramnion and prematurity became a hallmark. The seminal work of the Lifton and Hildebrandt groups showed that mutations in the genes that code for the apical transport in the TAL of Na^+^, K^+^ and Cl^−^ (NKCC2, ROMK2) as well as the basolateral extrusion mechanisms (ClC-Ka, Barttin) are essential for Na^+^ K^+^ and Cl^−^ handling in the TAL (Bartter’s Syndrome types 1–4) (28–33) Here, Mg^2+^ wasting is not caused by a direct mechanisms, and likewise Mg^2+^ wasting is a clinical hint, but not a mandatory feature of Bartter’s syndrome. Mg^2+^ wasting in Bartter’s Syndrome is also believed to be secondary to polyurias (and polydipsia) present in these patients. Thus, all the proteins are classified into Category 3 (Table 1) supported by the fact that also mice with a targeted deletion of NKCC2 show a Bartter-like phenotype but no hypomagnesemia (34). Interestingly, a transient form of Bartter’s syndrome has been described in several patients recently. Patients displayed the prenatal (Polyhydramnion, Prematurity) and postnatal (Hyponatrimia, Hypokalemia) clinical hallmarks of Bartter’s syndrome. The authors have shown that these disorders are caused by mutations in the gene encoding melanoma-associated antigen D2, explaining that fact that only males were affected (35, 36). Although the authors have not reported on Mg^2+^ levels, it is intriguing that this disorder is a more common cause of neonatal hypomagnesemia than anticipated that far.

The TAL is separated anatomically but also by its functional elements in the medullary and the cortical part (mTAL and cTAL). An important driving force in epithelial tissues and, therefore, also in the TAL, in order to maintain a transepithelial gradient is generated by the basolateral Na^+^ K^+^ ATPase. Mutations in the gene (FYXD2) coding for the γ-subunit of this protein have been shown to cause dominant familial Hypomagnesemia. Thus, it has been shown that this protein is a “conditio-sine-qua-non” for generating the necessary transepithelial gradient for transport systems and, thus, also for renal Mg^2+^ handling (11, 12, 37), this protein can be classified into the Category 2.

During the last years, several proteins have been shown to be involved in renal paracellular ion transport. However, a clear phenotype–genotype correlation has established in some but not in all of the genes and proteins involved. An example is provided by CLDN14. Mutations in CLDN14 cause non-syndromic deafness in men and mice (38, 39). Affected human individuals do not display overt renal abnormalities and the same holds true for mice with targeted deletions in CLDN14 (38). On the other hand, genome-wide association studies identified CLDN14 variants as a major risk gene associated with hypercalciuric stone disease (4, 40, 41). Furthermore, in the TAL, mutations in human genes (CLDN10, CLDN14, CLDN16, and CLDN19), that define the paracellular pathway (Figure 4) of Mg^2+^ absorption have been shown to cause rare human disorders. In the TAL, where significant Mg^2+^ and Ca^2+^ transcellular transport is absent, paracellular transport is of vital importance and is driven by a lumen-positive potential. Mutations in CLDN16 cause an autosomal-recessive disorder called “Familial Hypomagnesemia with Hypercalciuria and Nephrocalcinosis” (FHHNC) (42). Patients affected display renal Mg^2+^ and Ca^2+^ wasting accompanied by nephrocalcinosis. This disorder causes in most of the cases end stage renal disease, leading in many, but not all cases to the need for renal transplantation (43). A clinical significant problem is given by the fact that there currently is no general screening procedure at the neonatal stage or later for toddles and schoolchildren on hypercalciuria or hypermagnesemia. Thus, if such patients are referred to a secondary or tertiary center, respectively, chronic renal insufficiency and calcifications have often already progressed to a severe and often, irreversible state. In contrast to the human situation, mice with targeted deletion of CLDN16 recapitulate human renal Mg^2+^ and Ca^2+^ wasting, but do not show any signs of overt renal calcifications and, moreover, no signs of apparent or progressing renal insufficiency (44). There is currently no explanation for this significant difference; however, it is intriguing that solving this striking difference, a possible route for treatment of patients may be opened. A novel recent finding was that the absence of CLDN16 in ameloblasts explains the clinical finding of amelogenesis imperfecta in FHHNC patients and mice with CLDN16 deficiency (45). A similar finding was reported for patients with mutations in the genes coding for CLDN19 and similar for mice (CLDN3) (20, 46).

In 2006, Konrad et al. reported that mutations in human CLDN19 also lead to renal Mg^2+^ and Ca^2+^ wasting, clinically an almost phenocopy of patients with CLDN16 mutations (47). Although still a matter of debate, it is believed that CLDN16 and CLDN19 interact and form a heteromeric paracellular channel, with a cation selectivity including Ca^2+^ and Mg^2+^ (48) (Figure 4). However, they could also show that patients harboring CLDN19 mutations suffer from severe ocular involvement, as Claudin-19, but not Claudin-16 is expressed in the retinal pigment epithelium TJs, leading besides the renal Mg^2+^ and Ca^2+^ wasting to major vision problems (47, 49).

Another Claudin (Claudin-10) that contributes to Mg^2+^ handling exists at least in two principal forms. Claudin-10a and -10b, both confer different electrophysiological properties (anion-selective channel vs. cation-selective channel with a high preference for Na^+^) and their tissue distribution (Kidney and Uterus for Claudin-10a and ubiquitously for Claudin-10b) (50–52) (Figure 4). The expression of the Claudin-10b in almost every epithelial tissue might explain that mice with a targeted deletion die soon after birth. In sharp contrast, the generation of a kidney specific CLDN10-KO mouse (by the use of a ksp-Cre deleter strain) led to a vital mouse model. However, these mice displayed hypermagnesemia, hypocalciuria, nephrocalcinosis, and polyuria (53). Isolated mouse tubules of the TAL demonstrated a decreased paracellular Na^+^ permeability as well as higher expression of Claudin-16. Interestingly, recently four independent groups have reported mutations in human CLDN10 (54–57). Bongers et al. reported on two non-related patients presenting with alkalosis, hypokalemia, hypocalciuria, and hypercalcemia and a serum Mg^2+^ in the upper range of normal. They identified heterozygous mutations (P149R, Glu157_Tyr192del, and D73N) in two unrelated families. Hadj-Rabia et al. reported mutations in six patients from two unrelated families (S131L, M1T), resulting in an absence of CLDN10 at the plasma membrane. Affected members had high serum Mg^2+^ levels and renal loss of K^+^, Na^+^ and Cl^−^. Of interest is that patients also suffered from a variety of skin and teeth disorders (hypolacrymia, ichthyosis, xerostomia, and severe enamel wear). A similar renal phenotype was reported by Klar and colleagues (55). They identified a CLDN10 Mutation (N48K) in two distantly related families with 13 affected individuals presenting with anhidrosis and the inability to produce tears. Although serum levels of Na^+^ and K^+^ were in the normal range, all patients present with high Mg^2+^ serum levels. All groups identified homozygous or compound heterozygous mutations in CLDN10 and demonstrated thereby unequivocally the importance of Claudin-10 for human Mg^2+^ homeostasis. However, the recently described homozygote mutation (G163A) in a patient by Terliesner was reported to have normomagnesemia (57). So far, several different mutations in human CLDN10 have been shown to cause a renal tubular disorder that is characterized by hypokalemia, alkalosis, and hypermagnesemia. Moreover, as Claudin-10 is expressed in skin tissues, and several different symptoms of disordered dermal Na^+^ homeostasis could, therefore, be attributed to this defect.

Interestingly, the full CLDN10 knock-out mouse dies a few hours after birth, indicating that one or more organs different than the kidney and skin must be, if deficient for Claudin-10, vital for survival. Thus, the fact that human mutation does not lead to a lethal phenotype are intriguing in terms of compensatory mechanisms. However, the fact that Claudin-10 is expressed in the lung leads to spectate that the primary cause of postnatal death is caused by the absence of Claudin-10 in the lung (58, 59).

A corresponding mouse model has been generated by Breiderhoff and colleagues, by crossbreeding Claudin-16-deficient mice with a kidney-specific Claudin-10-deficient mouse strain (44, 53). Combining a hypomagnesemic model (CLDN16^−^/^−^) with a hypermagnesemic model (kidney specific CLDN10^−/−^) resulted in a normomagnesemic mouse, thus a “restored” normal phenotype (60). These findings point at the high compensatory, and more than so far anticipated capacity of the DCT. Put in perspective, the development of a selective renal Claudin-10b blocking agent could be a therapeutic option for Claudin-16 patients, since obviously the block of a Na^+^ pore restores the capacity of Mg^2+^ and Ca^2+^ recovery more distantly, i.e., the DCT.

### Distal Convoluted Tubule

In the DCT, Mg^2+^ reabsorption takes place mainly by transcellular route. Here, apical uptake, intracellular buffering, transport, and the extrusion at the basolateral site is concerted by a highly defined and regulated (e.g., hormonal) molecular machinery that has been recently reviewed in Ref. (5, 27). Among the eight different types of Mg^2+^ transport factors identified in eukaryotes (TRPM6/M7, Mrs2, MMgT, MagT1, SLC41 family, NIPA, HIP14, and CNNMs) (61–67), only three are expressed at the DCT. The selected list includes (1) the transient receptor potential channel melastatin member 6 (TRPM6) (68), (2) the third member of the solute carrier SLC41A family (SLC41A3) (69), and (3) the “Cyclin and CBS Domain Divalent Metal Cation Transport Mediator-2” (CNNM2), also referred to as ACDP2 (ancient conserved domain protein-2) (63, 65) (Figure 4).

These three proteins are classified in the first category of Table 1 as they are directly involved in Mg^2+^ transport or have been related to direct Mg^2+^ handling in the DCT.

TRPM6 was the first molecularly identified protein involved in active Mg^2+^ reabsorption (68). This channel, which associates in homotetramers, and may also interact with its closest homolog TRPM7 to form heterotetrameric species (70), is five times more permeable to Mg^2+^ than to Ca^2+^, and permits the reabsorption of these cations through the apical membrane of the epithelial cells (68). The three-dimensional structure of TRPM6 still remains unsolved, but its domain distribution is known and includes a cytosolic N-terminus followed by a transmembrane region of six α-helices and a long intracellular C-terminus that contains a serine-threonine active kinase domain similar to that present in α-kinases. The ion pore is putatively located between the fifth and sixth α-helices of the transmembrane section (71). Interestingly, mutations that impair the phosphorylation of threonine at position 1851 (72) decrease the protein transport activity. These findings have led to propose that autophosphorylation is a key step in the regulatory mechanism of Mg^2+^ transport through this channel (73). Clinical or genetical disturbances in TRPM6 are linked to different diseases. For example, mutations in its amino acid sequence cause the rare autosomal-recessive familial hypomagnesemia with secondary hypocalcemia (74–76). Other variants have been linked to hypoparathyroidism (77) and breast cancer (78). Of note, genetic ablation of the TRPM6 gene in mice results in early embryonic lethality (79). Interestingly, TRPM6^±^ mice showed reduced expression of the channel in kidney and colon, resulting in mild hypomagnesemia with no hypocalcemia (80).

A set of other mutations in genes that are not primarily associated with Mg^2+^, but relevant for the establishment of the apical membrane potential to drive Mg^2+^ entry through TRPM6 (and, therefore, classified in categories 2 and 3 in Table 1), have been identified causing secondary Mg^2+^ wasting. The corresponding genes code for transcriptional factors (HNF1B and PCBD1), growth factors (EGF), (co)-transporters (NCC, encoded by gene SLC12A3), or even ion-channels (Kir4.1 encoded by KCNJ10) (66, 81–84) (Figure 4). According to the gene mutated, the resulting phenotype comprises cystic kidney disease, diabetes, electrolyte disturbances other than Mg^2+^, or seizures. Clinically, hypomagnesemia is, compared to coexisting diabetes (MODY5), the chronic kidney disease and the hyperphenylinaemia a subordinated problem to the patient. However, identifying hypomagnesemia might be of value to identify the comorbidities at an early stage (85). Furthermore, elucidating the mechanisms that contribute to the disordered handling of Mg^2+^ in these patients may also enable a better understanding and treatment of diabetes, cyctic kidney disease, and hyperphenylinemia.

Inactivating mutations in SLC12A3 cause Gitelman syndrome, the most frequent cause of hereditary hypomagnesemia and characterized by hypokalemic metabolic alkalosis with hypomagnesemia and hypocalciuria. It has been proposed that a decrease activity of the NCC protein affects the membrane potential necessary for Mg^2+^ reabsorption in the apical membrane of DCT by TRPM6 (86–89).

SLC41A3 was originally described by Quamme as part of the solute carrier family 41 (65), which encompasses three integral cytoplasmic membrane putative Mg^2+^ transporters (SLC41A1, -A2, and -A3) (90). Mutations in this family are linked to Parkinson’s disease (91), diabetes (92), and nephrolithiasis (93). As in the case of TRPM6, the three-dimensional structure of these transporters remains unsolved, but is known to be built up of 10 or 11 transmembrane α-helices (94). SLC41A3, whose molecular function and interaction partners remain also elusive, is the highest enriched member in the DCT. Recently, de Baaij et al. found that, a Slc41a3^−/−^ knockout mice suffer from hypomagnesemia and normomagnesiuria, accompanied by upregulation of TRPM6 and SLC41A (69). These results underlined the relevant role played by SLC41A3 in Mg^2+^ reabsorption.

The third identified Mg^2+^ transport mediator expressed in the DCT is CNNM2, which belongs to the Cyclin M family. This family encompasses four different members (CNNM1-4). Mutations in CNNM2 cause dominant familial hypomagnesemia (67), and have been linked to brain development anomalies (95), hypertension, diabetes, and obesity (96, 97). Moreover, the CNNM2 locus has been linked by GWAS to neuro-psychiatric disorders (e.g., Schizophrenia) (98, 99). In 2014, Arjona et al. found that knockdown of CNNM2 orthologs in zebrafish results in brain abnormalities, increase of spontaneous contractions, and Mg^2+^ waste (95). These authors also identified five new families with mutations in CNNM2 that suffered hypomagnesemia with mental retardation and seizures. These findings suggested an essential role of CNNM2 in Mg^2+^ homeostasis and brain development. The relevant role of CNNM2 is underlined by the fact that mice lacking CNNM2 are embryonic lethal (100). Heterozygous (Cnnm2^+/−^) mice show lower Mg^2+^ levels in serum, thus suggesting defects in Mg^2+^ reabsorption in kidney. In addition, these animals showed lower blood pressure than compared to control mice. These results highlighted the importance of Mg^2+^ and its reabsorption in the kidney to maintain blood pressure (100).

The CNNMs represent the least-studied members across the mammalian transporters and share with MgtE and with the CLC family of chloride channels the presence of a cystathionine β-synthase (CBS) domain pair in their amino acid sequence (101–103). The four CNNM family members were first identified in 2003 by Wang et al. (61, 104) and show very strong homology to the bacterial CorC protein [which is involved in Mg^2+^ and cobalt (Co^2+^) efflux (63)], and with the Mam3p proteins (67, 105). It was initially suggested that CNNMs might be involved in cell-cycle regulation (61), as they contain a cyclin box-like motif and are located in the plasma membrane. However, the cyclin M function has that far not been proved *in vivo*.

**Figure 5:**
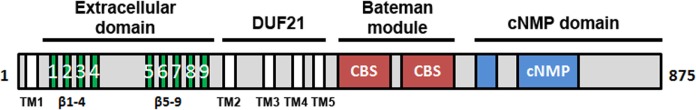
Domain distribution of CNNM2. CNNM2 is a 97 kDa protein of 875 amino acid residues, formed by four different domains: (i) the N-terminal extracellular region is enriched in β-strands and presents a large cleavage signal peptide of approximately 65 residues. This zone is preceded by a transmembrane α-helix (TM1); (ii) the following DUF21 domain includes 3 or 4 transmembrane helices (TM1-4) that are putatively responsible for Mg^2+^ transport across the membrane; the intracellular part includes two domains: (iii) a Bateman module consisting of two consecutive CBS domains and (iv) a cyclic nucleotide monophosphate (cNMP)-like domain. CNNM2 shows two isoforms (Iso1 and Iso2). Iso2_CNNM2 lacks residues 721–742.

The second member of the Cnnm family, CNNM2, is abundant in brain and kidney (64, 65), and shows a complex modular architecture composed by four structural domains (Figure 5) (106). The N-terminal section (likely to be an extracellular compoment) consists of a β-stranded enriched region (residues 1–250) and precedes a DUF21 domain (residues 251–400, Pfam code PF01595) with three or four transmembrane α-helices (107). The following intracellular region includes a CBS domain pair (so called “Bateman” module; Pfam code PF00571) (106) and a C-terminal cyclic nucleotide monophosphate (cNMP) like binding domain (Pfam code PF00027) (106) (Figure 5). Although the concrete function of each domain remains unknown, recent biophysical and structural data supports a regulatory role for the Bateman module.

### Mechanisms of Mg^2+^ Transport in the DCT and CNNM2

Despite the universally recognized relevance of magnesium in maintaining key life processes as mentioned above, current knowledge about the CNNMs and their role in the DCT, as well as the molecular mechanisms involved in its transport across the cellular membranes remain still to be explored. This is largely due to the scarce structural information available on Mg^2+^ transporters and channels, that so far was limited to the crystal structures of two prokaryotic proteins: (i) CorA from *Thermotoga maritima* (108–111) and (ii) MgtE from *Thermus thermophilus* (112–115). These proteins are homologs of two eukaryotic Mg^2+^ transport mediators: the mitochondrial Mg^2+^ channel Mrs2 and the solute carrier (SLC) family 41 members (homologs of CorA and MgtE, respectively). CorA represents the major transport machinery responsible for Mg^2+^ uptake in bacteria and it translocates Mg^2+^ by using an inwardly biased electrochemical gradient that serves as the driving force for Mg^2+^ permeation (108, 109, 111, 116).

MgtE is a dimeric Mg^2+^ selective channel (117) that permeates Mg^2+^ ions and maintains the intracellular Mg^2+^ homeostasis in bacteria. MgtE shares with CNNM2 (but not with SLC41) the presence of an intracellular CBS domain pair (101, 102). An ATP/Mg-dependent open-to-close gating process that involves binding of the nucleotide at this region defines the threshold of intracellular Mg^2+^ for the channel inactivation and provides sensory capacity to this protein (113, 114, 118).

**Figure 6:**
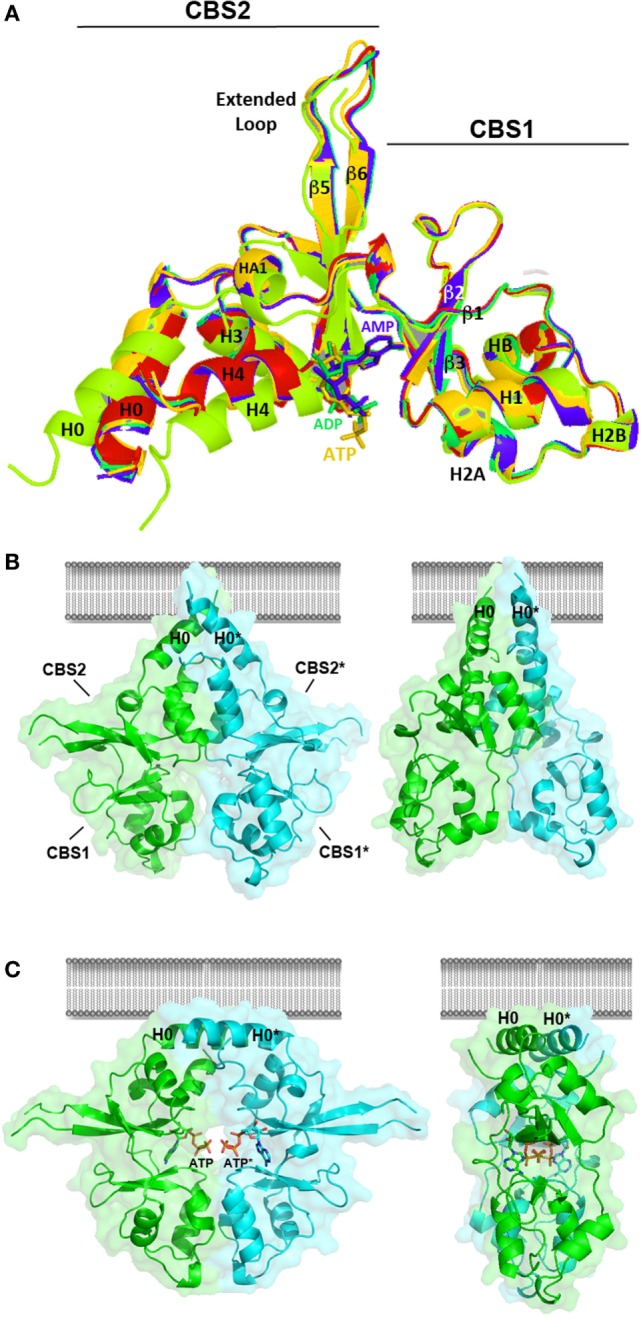
Crystal structure of the Bateman module of CNNM2. **(A)** The Bateman module of CNNM2 consists of two consecutive cystathionine β-synthase (CBS) motifs (CBS1, residues 445–508; CBS2, residues 509–578). A long extended loop links strands β5 and β6 in the CBS2 motif. The H0 helix connects CBS1 with the DUF21 transmembrane domain in the full-length protein. The H4 helix connects CBS2 with the cyclic nucleotide monophosphate domain. Nucleotides, ca. AMP (blue), ADP (green), or adenosine triphosphosphate (ATP) (orange) bind independently at the S2 site, thus disrupting the interactions formerly existing in the cavity between residues of the CBS1 and CBS2 motifs. This induces a displacement of helices H0, H1, and H4 in each Bateman subunit. The apo- and the nucleotide-bound Bateman module are represented in green and marine, respectively. The crystal structure of the T568I protein variant is in red. As shown, the T568I mutation mimics the structural effect of ATP binding; although in the first case, the structural change is irreversible, thus locking the protein in the nucleotide-bound like conformation. **(B,C)** *Conformational changes induced by ATP*. The Bateman module of CNNM2 associates in disk-like dimers known as “CBS modules,” which adopt a *twisted* **(B)** or a *flat* **(C)** state depending on whether the site S2 of each subunit is empty or hosts an ATP (or MgATP) molecule, respectively. Note that the H0 helices connecting the CBS2 motif with the DUF21 domain are differently oriented in each case, thus likely transmitting the transformation suffered by the Bateman module to the transmembrane region.

The recent elucidation of the crystal structure of the Bateman module of CNNM2 (119, 120) has shed new light on the molecular mechanisms underlying Mg^2+^ transport through the basolateral membrane of the DCT (Figure 6). We and others recently confirmed that this region may host ATP in a Mg^2+^-dependent manner, as well as independent Mg^2+^ atoms that interact with some acidic clusters located in the CBS1 motifs (106, 119). The fact that the Mg^2+^ independent sites are far away from the nucleotide suggest that, as observed in MgtE (114), binding of Mg^2+^ at concrete positions may not be directly coupled with ATP binding, although it may complement its effect in conformational transformations suffered by the whole module (106, 121).

The Bateman module of CNNM2 features two major cavities (named as S1 and S2) that are located at opposite ends of the central β-sheets of the CBS motifs. In contrast with S1, which is partially occluded and full of bulky residues, the site S2 is fully accessible and can accommodate phospho-nucleotides, such as AMP, ADP, or ATP (106, 121) (Figure 6). Site S2 is built by three different structural blocks: (i) the central residues from the linker preceding the first β-strand (β1) of the CBS1 domain, (ii) the C-terminal residues from the last β-strand (β6) of the CBS2 motif, and (iii) the first two turns of helix H4 of CBS2 (Figure 6). The upper and right walls of the cavity are mainly hydrophobic and help accommodating the bulky adenine ring of ATP (106, 121). By contrast, the left wall of the cleft is hydrophilic and is built from the last β-strand and the following α-helix (H4) of the CBS2 motif. A conserved threonine (T568) and an aspartate residue (D571) from this helix are key in help orienting the ribose ring of the nucleosides inside the cavity, and if mutated, impede the allocation of ATP inside (106). Interestingly, the repulsive effect otherwise exerted by the acidic cluster formed by residues E570, D571, and E574 (at the first turn of α-helix H4 of CBS2) over the polyphosphate chain of ATP (106) is neutralized by the Mg atom that accompanies the ATP molecule. The positive dipole end of helix H4 and the nearby arginine residue, R480, complement the neutralizing effect (106). The Bateman module of CNNMs associates in head-to-head oriented disk-like dimers known as *CBS modules* (Figures 6B,C) (106, 121).

**Figure 7:**
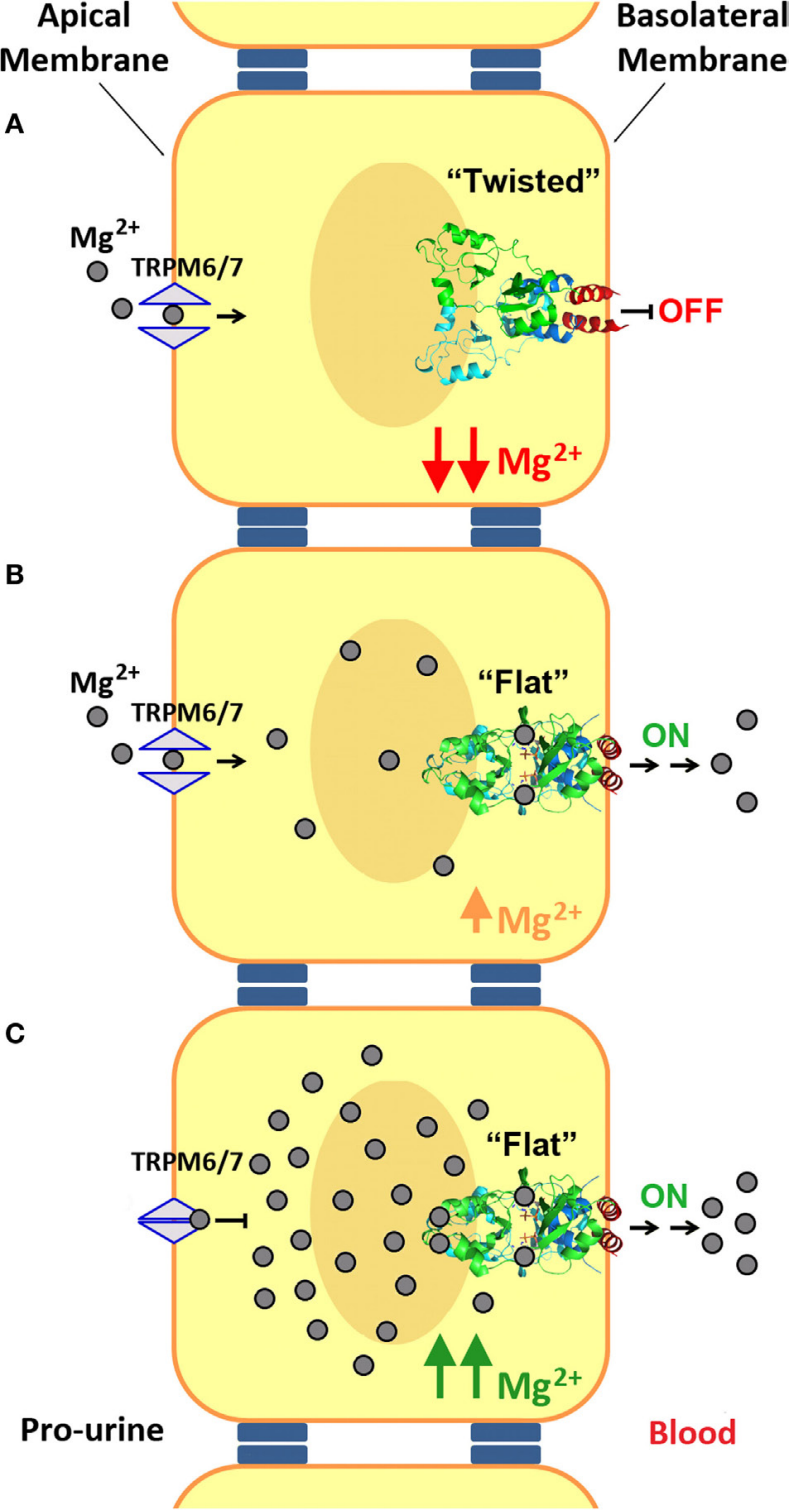
Mechanism of Mg^2+^ transport at the distal convoluted tubule (DCT). **(A)** Mg^2+^ enters into the DCT epithelial cells through the apical membrane with the help of TRPM6/7 channels. At low Mg^2+^ concentrations, the cystathionine β-synthase (CBS) module of CNNM2, located at the basolateral membrane, remains in its twisted conformation. **(B,C)** Upon increasing the intracellular concentration of Mg^2+^, binding of these cations and of MgATP to CNNM2, triggers the progression of the CBS module toward its flat state, and the transport of Mg^2+^ through the basolateral membrane toward the blood torrent. In addition, an increased intracellular Mg^2+^ concentration inhibits apical transport by TRPM6.

At low concentrations of Mg^2+^ and in the absence of MgATP, the CBS module adopts a “twisted” shape (Figure 6B), in which the CBS2 domains from complementary subunits remain in contact while the CBS1 motifs are separated and retain only scarce hydrophobic interactions. Binding of MgATP at site S2 disrupts a network of H-bonds centered on the conserved threonine at position 568 (T568) as well as a salt bridge between R480 and E570 and causes the displacement the C-terminal helix of the CBS2 domain (helix H4) as well as of the long α-helix (helix H0) that connects the Bateman module with the DUF21 domain (Figure 5). These structural changes, which occur concomitantly in the two complementary subunits of the dimer, trigger an overall rearrangement of the CBS module that makes it to evolve from a “twisted” (Figure 6B) toward a “flat” disk structure (Figure 6C). The conformational effect of ATP is likely transferred to the DUF21 transmembrane domain through helix (H0) that connects it with the CBS2 motif. This sequence of events has been postulated as the mechanism by which CNNM2 might regulate the gating of Mg^2+^ ions through the basolateral cell membrane (106, 121) (Figure 7).

### CNNM2 and MgtE Differ in Their CBS-Domain-Mediated Gating Mechanism

Interestingly, an ATP/Mg-mediated gating process ruled out by CBS domains has also been postulated as the mechanism by which the MgtE transporter senses and regulate the Mg^2+^ homeostasis in bacteria (114). In contrast with the twisted-to-flat transformation observed in CNNM, the rearrangement of the Bateman modules of MgtE responds to an open-to-close mechanism (113–115). In the absence of Mg^2+^ ions, the complementary CBS2 motifs of MgtE subunits remain apart in the dimer due to the repulsion exerted by acidic clusters located at the interfacial helices of the CBS2 domains. In this state, the CBS module impairs the transport of Mg through the membrane and maintains the protein in an “open” state. While the intracellular concentration of Mg^2+^ is low, the CBS module remains open and allows the influx of Mg^2+^ ions toward the interior of the cell. Upon increasing the intracellular concentration of Mg^2+^, the pre-existing repulsive acidic clusters become sequentially neutralized by newly bound Mg^2+^ ions, thus allowing the approximation of the CBS2 motifs. The sequential binding of Mg atoms progressively causes a closure of the CBS module that, when completed, adopts a “flat” disk-like arrangement as that observed in the MgATP/Mg^2+^ bound form of CNNM2 (106). The new state is transferred to the transmembrane region and results in the closure of the membrane pore (114).

## Conclusion

The identification of mutations in human genes has led to a deeper understanding of Mg^2+^ handling in health and disease. On that basis, technologies, such as mouse genetic engineering as well as crystallography, have contributed in this field of physiology and pathophysiology. However, our current knowledge of the molecular mechanisms underlying magnesium transport through the cell membranes is very scarce, and represents an incipient field of research that will mature, as we are able to identify new molecular partners involved in this process. Proteins involved in trans- and paracellular pathways have the potential of being key players in Magnesium Homeostasis and also other disorders, such as diabetes, hypertension, and schizophrenia, thus being a potential target for pharmaceutical interventions.

## Author Contributions

All authors listed have made a substantial, direct, and intellectual contribution to the work and approved it for publication.

## Conflict of Interest Statement

The authors declare that the research was conducted in the absence of any commercial or financial relationships that could be construed as a potential conflict of interest.
